# Copper-induced diurnal hepatic toxicity is associated with *Cry2* and *Per1* in mice

**DOI:** 10.1265/ehpm.23-00205

**Published:** 2023-12-13

**Authors:** Sarah Tominaga, Hiroki Yoshioka, Satoshi Yokota, Yosuke Tsukiboshi, Masumi Suzui, Makoto Nagai, Hirokazu Hara, Tohru Maeda, Nobuhiko Miura

**Affiliations:** 1 College of Pharmacy, Kinjo Gakuin University, 2-1723 Omori, Moriyamaku, Nagoya, Aichi 463-8521, Japan; 2 Department of Neurotoxicology, Nagoya City University Graduate School of Medical Sciences, 1 Kawasumi, Mizuho-cho, Mizuho-ku, Nagoya, Aichi 467-8601, Japan; 3 Faculty of Pharmacy, Gifu University of Medical Science, 4-3-3 Nijigaoka, Kani, Gifu 509-0293, Japan; 4 Division of Cellular and Molecular Toxicology, Center for Biological Safety and Research, National Institute of Health Sciences, 3-25-26 Tono-machi, Kawasaki-ku, Kawasaki, Kanagawa 210-9501, Japan; 5 Graduate School of Health and Medicine, Gifu University of Medical Science, 795-1 Nagamine Ichihiraga, Seki, Gifu 501-3892, Japan; 6 Laboratory of Clinical Pharmaceutics, Gifu Pharmaceutical University, 1-25-4 Daigaku-nishi, Gifu, Gifu 501-1196, Japan; 7 Department of Health Science, Yokohama University of Pharmacy, 601 Matano-cho, Totsuka-ku, Yokohama, Kanagawa 245-2006, Japan

**Keywords:** Copper, Chronotoxicity, Hepatotoxicity

## Abstract

**Background:**

This study aimed to investigate diurnal variations in copper-induced hepatic toxicity and the molecular mechanisms underlying this chronotoxicity.

**Methods:**

Male C57BL/6J mice were intraperitoneally injected with copper chloride (CuCl_2_) at zeitgeber time 2 (ZT2) or 14 (ZT14), twice per week for 5 or 8 weeks. Seventy-two hours after the final CuCl_2_ injection, the mice were euthanized, and plasma samples were collected. The livers and kidneys were collected and weighed. *In vitro* experiments were performed to assess cell viability and fluctuations in clock gene expression levels in Hepa1-6 cells after CuCl_2_ treatment. We examined copper homeostasis- and apoptosis-related genes under clock genes overexpression.

**Results:**

Repeated CuCl_2_ administration for 8 weeks resulted in more severe toxicity at ZT14 compared to ZT2. CuCl_2_ administration at ZT14 elevated plasma aspartate aminotransferase, hepatic *tumor necrosis factor-α*, and *interleukin-6* for 5 weeks, whereas the toxic effects of CuCl_2_ administration at ZT2 were weaker. Moreover, CuCl_2_ treatment inhibited Hepa1-6 cell viability in a dose-dependent manner. We observed increased expression of three clock genes (*Ciart*, *Cry2*, and *Per1*) after CuCl_2_ treatment. Among them, overexpression of *Cry2* and *Per1* accelerated CuCl_2_-induced inhibition of Hepa1-6 cell viability. Moreover, we found that the overexpression of *Cry2* and *Per1* regulates cleaved caspase-3 by modulating the copper transporter genes ATP7B and CTR1.

**Conclusion:**

These results suggest that CuCl_2_-induced diurnal toxicity is associated with *Cry2* and *Per1* expression through the regulation of copper transporter genes in mice.

**Supplementary information:**

The online version contains supplementary material available at https://doi.org/10.1265/ehpm.23-00205.

## Graphical Abstract

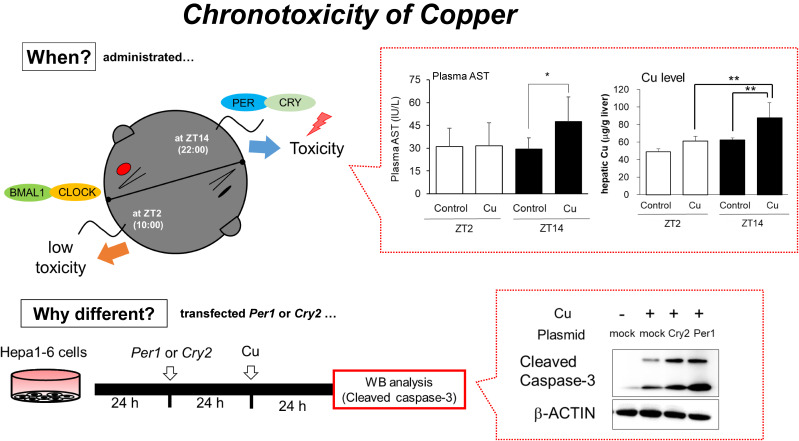

## Background

Shift work is a common practice in various industries and services, including steel factories, power plants, nursing, and police forces, in which professionals work during both day and night shifts [1]. In the United States of America, approximately 20–30% of employees are categorized as shift workers [2]. However, shiftwork has been associated with various health issues, such as cancer, obesity, and cardiovascular disease, due to disruptions in circadian rhythms [3–6]. Factors contributing to circadian rhythm disruption include light exposure, specific diets (i.e., high-fat diet), chemical exposures, and radiation exposures [7–9]. Therefore, preventing unexpected circadian rhythm disorders is essential.

Copper (Cu) is an essential cofactor for key metabolic enzymes involved in various physiological processes, including radical detoxification, iron uptake, respiration, and neurotransmitter biosynthesis [10]. However, free Cu ions are potentially toxic to cells due to their redox activity [11]. Mutations in Cu-binding proteins or Cu overdose have been correlated with diseases such as Alzheimer disease, Wilson disease, and Menkes disease [12, 13]. Various studies have reported that accumulated Cu disrupts cellular homeostasis, leading to oxidative stress and apoptosis in the liver, brain, kidneys, and spleen [14, 15]. Since excessive Cu exposure can occur due to accidents or occupational hazards, proper Cu handling is crucial for workers in the steel industry.

We studied the relationship between the timing of medicine or chemical administration and the severity of toxicity as chronotoxicology [16–21]. For instance, we previously demonstrated that the industrial solvent bromobenzene induces more severe toxicity during the light phase than during the dark phase [17]. Bromobenzene metabolites, such as 4-bromocatechol, confirmed renal dysfunction during the dark phase [19]. Additionally, we evaluated the chronotoxicology of seven metals (Hg, Pb, Ni, Cr, Cu, Zn, and Fe) [18] in mice. The mice were susceptible to Cu and Zn toxicity when the light period changes to the dark period. Moreover, we recently reported that overexpression of *Period2* (*Per2*) and *neuronal PAS domain protein 2* (*Npas2*) attenuates Zn-induced toxicity in murine hepatoma Hepa1-6 cells [21]. However, limited information is available on the chronotoxicity of Hg, Pb, Ni, Cr, Cu, and Fe, as we only tested for their lethal toxicity [18]. Therefore, further research is necessary to elucidate the chronotoxicity related to metal exposure.

In this study, we examined diurnal variations in Cu-induced hepatic toxicity and investigated the biological factors involved in this chronotoxicity in mice.

## Methods

### Animal experimental protocol

Forty male 6-week-old C57BL/6J mice were purchased from CLEA Japan, Inc. (Tokyo, Japan) and housed in a controlled environment (temperature 24 ± 1 °C and humidity 55 ± 5%) with a standard 12-h light/dark cycle (8:00, zeitgeber time 0 [ZT0]; 20:00, ZT12) and provided with food and water *ad libitum*. This experiment was approved by the Institutional Animal Care and Experimentation Committee of Kinjo Gakuin University and Gifu University of Medical Science. After 1 week of acclimatization to laboratory conditions, the mice were randomly divided into four groups (10 mice each): the control group at ZT2, Cu-treated group at ZT2, control group at ZT14, and Cu-treated group at ZT14. Copper chloride (CuCl_2_: Fujifilm Wako Pure Chemical, Osaka, Japan) was intraperitoneally injected into the mice at 6.08 mg/kg (45 µmol Cu/kg) at 10:00 (ZT2) or 22:00 (ZT14) twice per week (Monday and Thursday) for 8 weeks, while the control group received intraperitoneal injections of saline solution as a placebo.

The mice’s body weight was measured weekly during the study period. After the final CuCl_2_ injection (Monday), the mice were euthanized 72 h later (Thursday), and plasma samples were obtained by cardiocentesis and stored at −80 °C. The livers and kidneys were weighed, and liver samples were either snap-frozen in liquid nitrogen and stored at −80 °C or fixed in 15% neutral-buffered formalin (pH 7.4; Fujifilm Wako Pure Chemical).

### Plasma biochemical analysis

Plasma alanine aminotransferase (ALT) and aspartate aminotransferase (AST) activities were measured using the Transaminase CII Test (Fujifilm Wako Pure Chemical) according to the manufacturer’s instructions and our previous studies [22–24]. Calibration curves were prepared using standard solutions for relative quantification.

### Quantitative RT-PCR

Total RNA from liver sections (80 mg) and Hepa1-6 cells were extracted using ISOGEN II (Nippon Gene, Tokyo, Japan), or RNA Basic Kit (NIPPON Genetics, Tokyo, Japan), respectively, following the manufacturer’s protocol (n = 3–6). The levels of each target mRNA were normalized to those of β-actin. Oligonucleotide sequences of the primers are listed in Supplementary Table S1.

### Histopathological analysis

A portion of the left lobe of the liver from each animal was perfused with phosphate-buffered saline (PBS) (pH 7.4) and neutral buffered formalin, dehydrated, and embedded in paraffin. Tissues were sliced into 4 µm-thick sections and stained with hematoxylin and eosin (H&E), following previous protocols [25, 26].

### Cell viability assay

Murine hepatoma Hepa1-6 cells obtained from the RIKEN Cell Bank (Tsukuba, Japan) were cultured in Dulbecco’s modified Eagle’s medium (high glucose) (DMEM; Fujifilm Wako Pure Chemical) supplemented with 10% fetal bovine serum and penicillin/streptomycin (Fujifilm Wako Pure Chemical) at 37 °C in a humidified atmosphere with 5% CO_2_. Hepa1-6 cells were seeded into 96-well plates at a density of 10 000 cells/well and treated with various concentrations of CuCl_2_ (Fujifilm Wako Pure Chemical) 24 h after seeding. After 24 h of Cu treatment, the cell viability was assessed using Alamar Blue (Bio-Rad Laboratories, Hercules, CA). For the transfection experiment, Hepa1-6 cells were seeded into 96-well plates at a density of 10 000 cells/well and transfected with 50 ng of pcDNA3.1-neo, pcDNA3.1-neo-circadian associated repressor of transcription (*Ciart*), pcDNA3.1-neo-cryptochrome 2 (*Cry2*), or pcDNA3.1-neo-*Per1* using the TransIT-LT1 Transfection Reagent (Takara Bio) after 3 h of seeding. After 24 h of transfection, cells were treated with 500 or 1000 µM Cu, and cell numbers were evaluated after 24 h of Cu treatment. The progress of plasmid construction is described in the Supplementary Fig. S1.

### Western blot analysis

Hepa1-6 cells were plated at a density of 500 000 cells per 35 mm dish and transfected with 1 µg pcDNA3.1-neo, pcDNA3.1-neo-*Cry2*, or pcDNA3.1-neo-*Per1* using the TransIT-LT1 Transfection Reagent after 24 h of seeding. Twenty-four hours after transfection, the cells were treated with 500 µM Cu or MilliQ water (0.1%). After 24 h, the cells were homogenized in 100 µL of ice-cold RIPA buffer (Nacalai Tesque) containing a protease inhibitor and centrifuged (18000 × *g* for 20 min at 4 °C). The supernatant from each sample was collected and protein was extracted using a BCA protein kit (Nacalai Tesque). The concentration of each protein samples was normalized to 1.0 µg/µL by adding 6x sample buffer (Nacalai Tesque) and 2-mercaptoethanol (355 mM: Fujifilm Wako Pure Chemical). After heat denaturation (100 °C, 3 min), protein samples (10 µg) were separated by 10% SDS-PAGE and transferred to a polyvinylidene fluoride membrane. The primary antibodies used were as follows: mouse ATPase copper-transporting beta (ATP7B) monoclonal antibody (1:1000; Santa Cruz Biotechnology, Dallas, TX), mouse β-actin monoclonal antibody (1:2500; MBL, Aichi, Japan), rabbit cleaved caspase-3 polyclonal antibody (1:3000; Cell Signaling Technology, Beverly, MA) and rabbit copper uptake protein 1 (CTR1) polyclonal antibody (1:3000; Sigma-Aldrich). Peroxidase-conjugated anti-mouse IgG or anti-rabbit mouse IgG were used as the secondary antibody (1:5000; Cell Signaling Technology).

### Determination of liver copper concentration

Individual liver specimens (40–100 mg each) were digested in 0.5 mL of concentrated nitric acid in glass test tubes. The temperature was held at 90 °C for 1 h, then gradually increased (at 10 °C per h) to 130 °C. When the acid-digested specimens became transparent, volumes of the digests were raised to 5 mL with distilled water, and copper concentrations were determined by atomic absorption using a Z-2300 (Hitachi, Tokyo, Japan).

### Statistical analysis

Multiple comparisons were performed using a one-way analysis of variance (ANOVA) with Tukey’s test. All statistical analyses were performed using SPSS Statistics for Windows (version 24.0; IBM Corp., Armonk, NY). Differences were considered statistically significant at *P* < 0.05.

## Results

### Lethal toxicity test

To investigate Cu-induced chronotoxicity, we administered a single injection of CuCl_2_ (9.0 mg/kg) to C57BL/6J mice at six different time points (ZT2, ZT6, ZT10, ZT14, ZT18, or ZT22). Mice injected at ZT10 and ZT14 experienced 100% mortality within 5 days (Supplementary Fig. S2A). At ZT2, ZT6, and ZT22, 40% [2] of the mice died within 14 days after the injection. The ZT18 injection group, had an 80% mortality rate (Supplementary Fig. S2B). This result indicated that the mice were tolerant to Cu-induced toxicity at ZT2, ZT6, and ZT22, whereas highly susceptible at ZT10 and ZT14. This result aligns with our previous study in ICR mice [18].

**Fig. 1:**
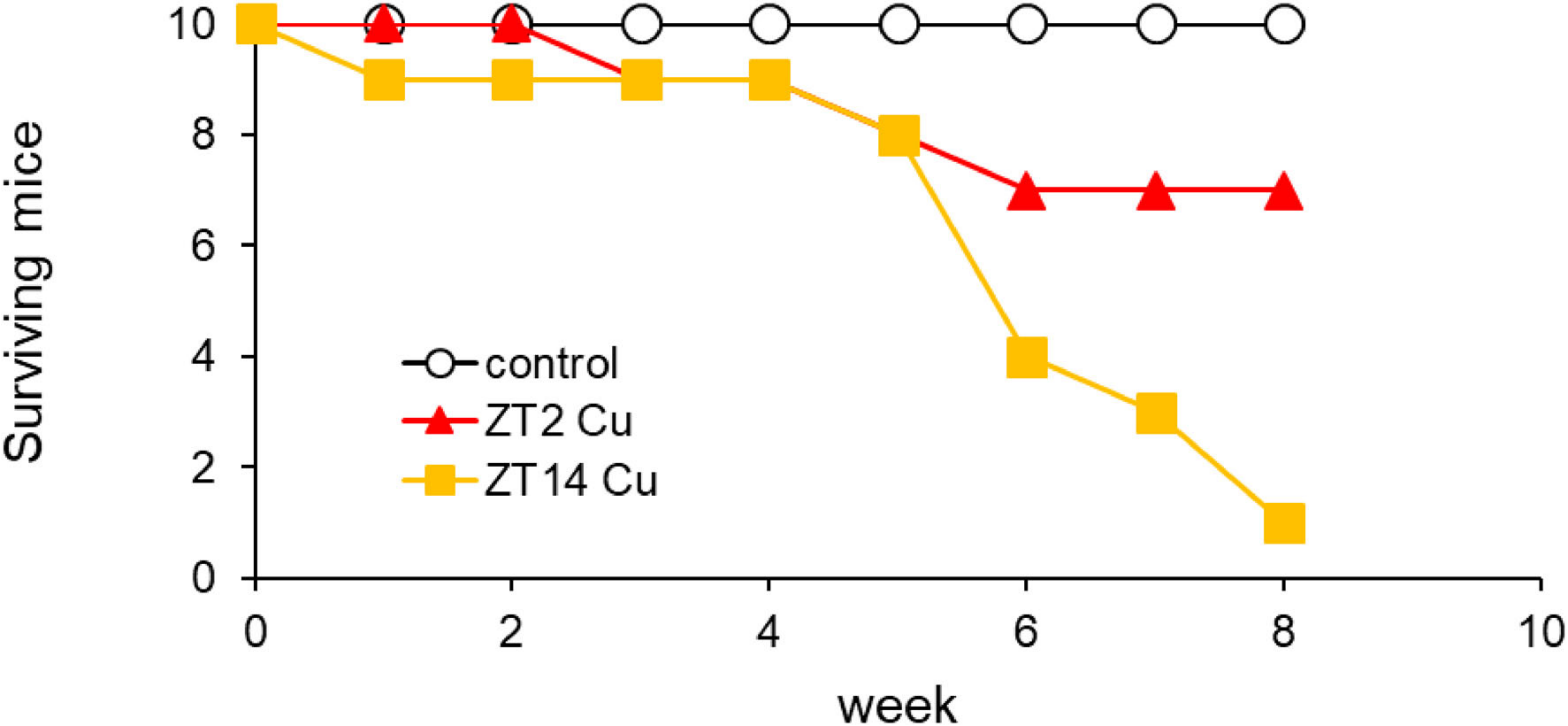
Survival numbers of mice after Cu repeated injection at ZT2 and ZT14 for 8 weeks. Male C57BL/6J mice (n = 10) were intraperitoneally injected with 6.08 mg/kg CuCl_2_ twice a week at ZT2 or ZT14 for 8 weeks.

Subsequently, we examined the repeated effects of Cu-induced chronotoxicity using a lower dose of CuCl_2_ (6.08 mg/kg). Based on the previous results, we selected ZT2 and ZT14 as representative injection times (Supplementary Fig. S2). Cu administration at ZT2 resulted in a gradual increase in mortality, with a 30% mortality rate (3/10) after 8 weeks (Fig. 1). In contrast, although a similar pattern was observed until 5 weeks, sudden death occurred at 6 weeks, leading to a 90% (9/10) mortality rate after 8 weeks. These data confirmed the conservation of Cu-induced chronotoxicity after single and repeated administrations.

### Body weight change and organ weight

We examined the effect of injection timing on the toxicity severity under non-lethal conditions during a 5-week administration, since mortality ratio between ZT2 and ZT14 remained the same after 5 weeks (Fig. 1). Throughout the experiment, exposure to Cu tended decrease body weight compared to the control group (Supplementary Fig. S3). Following Cu administration, the change in body weight was almost the same until 4 weeks. To examine the effects of Cu on organ weight, we measured the weights of liver and kidney samples (Table 1). The liver/body weight ratio of the control group at ZT2 was significantly higher than that at ZT14 (*P* < *0.01*). Because mice tend to eat at midnight, the liver weight at ZT2 decreased by glycogenolysis [27]. After Cu administration, no changes in the liver/body weight ratio were observed at ZT2, whereas a significant increase was observed at ZT14 (*P* < *0.05*) (Table 1, Supplementary Fig. S3). Similar results were observed in the kidneys, indicating that Cu exposure at ZT14 affected the Cu-induced increase in relative liver weight.

**Table 1:** Body weight, liver and kidney ratio in the mouse administrated with Cu for 5 weeks at ZT2 or ZT14

	**BW (g)**	**Liver/BW (%)**	**Kidney/BW (%)**
ZT2 control	25.82 ± 1.50	5.14 ± 0.17	1.21 ± 0.07
ZT2 Cu	23.84 ± 1.00	5.15 ± 0.36	1.22 ± 0.08
ZT14 control	25.59 ± 1.55	4.00 ± 0.13^a^	1.09 ± 0.03^c^
ZT14 Cu	23.06 ± 2.46	4.44 ± 0.46^b^	1.16 ± 0.06^b^

a: *P* < *0.01* versus ZT2 control, b: *P* < *0.05* versus ZT14 control, c: *P* < *0.05* versus ZT2 control.

### Plasma biochemical parameters and inflammatory cytokines

**Fig. 2:**
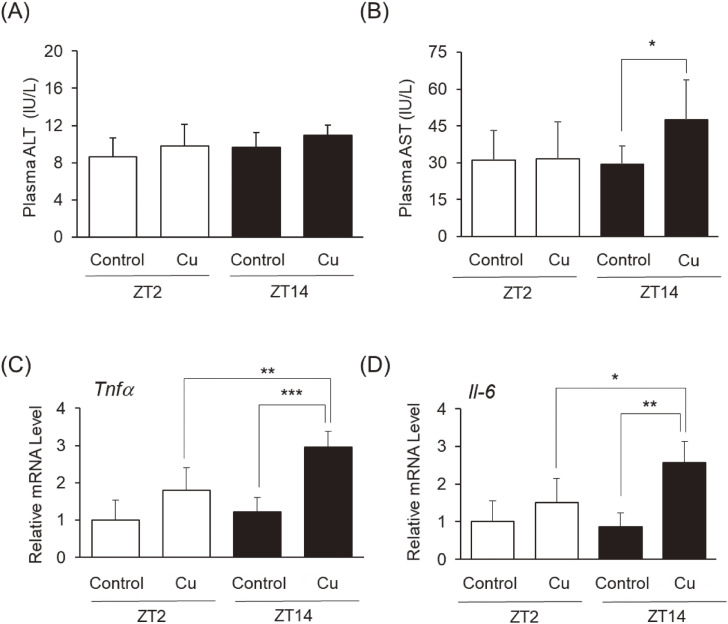
Effect of injection time against Cu-induced hepatotoxicity. Male C57BL/6J mice (n = 10) were intraperitoneally injected with 6.08 mg/kg CuCl_2_ twice a week at ZT2 or ZT14 for 5 weeks. Mice were euthanized 72 h after the final Cu injection. Panels (A) and (B) indicate the plasma ALT and AST levels. Panels (C) and (D) illustrate hepatic *Tnfα* and *Il-6* expression levels, respectively. Data represent the mean ± SD of 10 mice per group; *P < 0.05, **P < 0.01, and ***P < 0.001.

Chronic exposure to Cu induces toxicity in the liver through oxidative stress, cell death, and inflammatory responses [28]. As illustrated in Fig. 2, we measured plasma levels of ALT and AST, indicators of hepatic injury. ALT levels remained unchanged after Cu administration at both ZT2 and ZT14 (Fig. 2A). In contrast, Cu exposure at ZT14 significantly increased AST levels (Fig. 2B). In addition, we measured hepatic *tumor necrosis factor-α* (*Tnfα*) and *Interleukin-6* (*Il-6*), markers of inflammatory cytokines (Fig. 2C and 2D). Cu administration at ZT2 tended to increase each level, whereas Cu administration at ZT14 significantly upregulated *Tnfα* and *Il-6* in the liver compared to the control group at ZT14 and Cu at ZT2, respectively.

### Histopathology

**Fig. 3:**
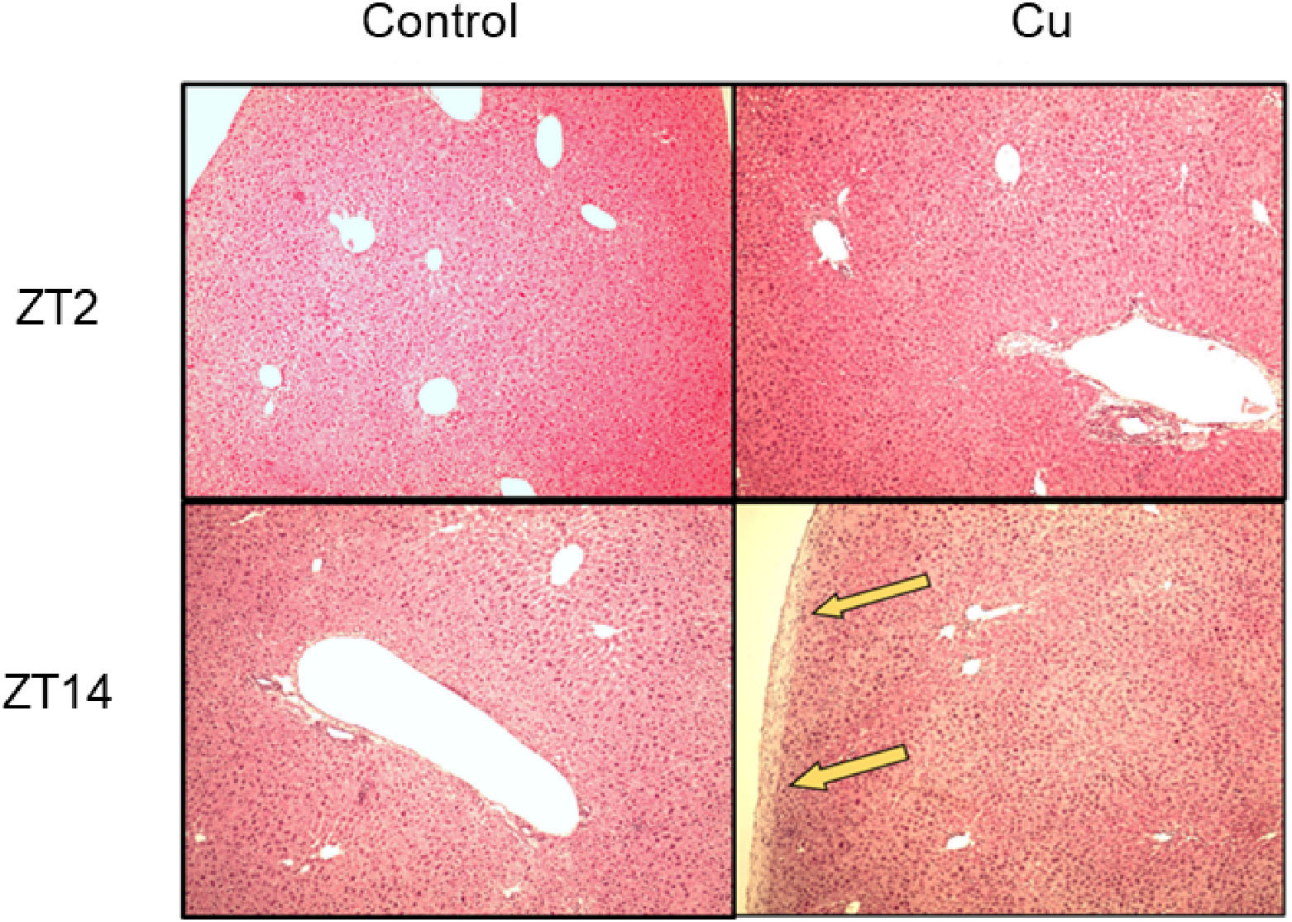
Cu injection at ZT14 worsened the hepatic morphology by H&E staining. The animals, treated as described in Fig. 2, were euthanized 72 h after intraperitoneal injection, and the livers were harvested during necropsy. Liver specimens were fixed, processed using standard methods, and stained with H&E.

Along with the measurement of plasma biochemical parameters and inflammatory cytokines, we conducted histopathological studies on liver tissues (Fig. 3). Liver sections of the control (at ZT2 and ZT14) and Cu-treated groups (at ZT2), stained with H&E, exhibited a normal hepatic architecture. In contrast, the livers of Cu-treated mice at ZT14 evidenced thickening of the liver capsules.

### Inhibition of cell viability and contribution of clock genes

**Fig. 4:**
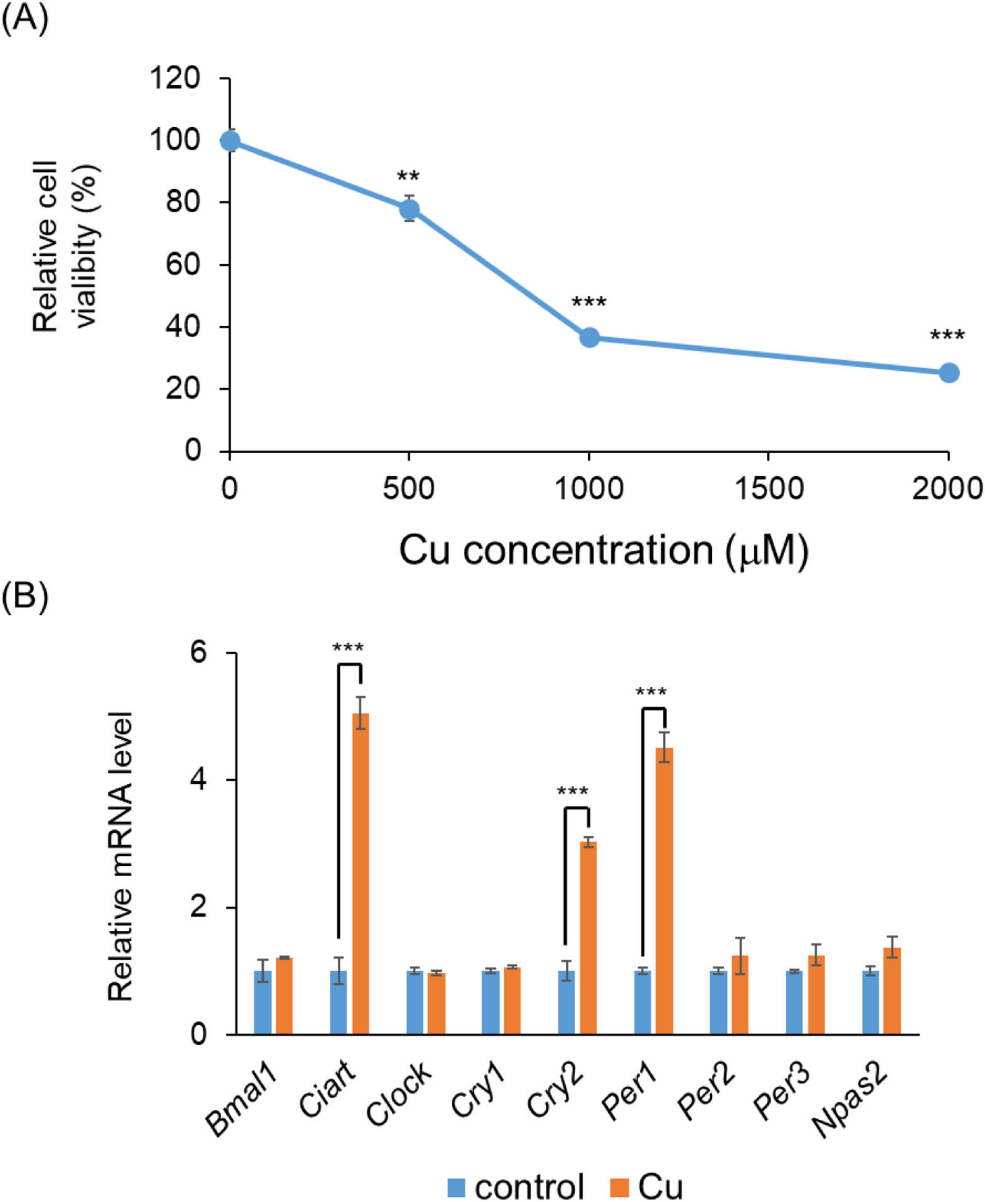
Influence of Cu treatment on cell viability and clock genes of Hepa1-6 cells (A) Hepa1-6 cells were seeded at a density of 10 000 cells into 96-well plates. After 24 h of cell seeding, cells were treated with Cu (500, 1000, and 2000 µM). The viability of cells was measured 24 h after the Cu treatment. Data are plotted as mean ± S.D. of groups **P < 0.01 and ***P < 0.001 versus control group (n = 6). (B) Hepa1-6 cells were plated at a density of 500 000 cells per 35 mm dish. After 24 h of cell seeding, cells were treated with 500 µM Cu. After 24 h of Cu treatment, the expression levels of clock genes were measured by RT-PCR. Data are plotted as mean ± S.D. of groups ***P < 0.001 versus control group (n = 4).

To investigate the mechanism of Cu-induced diurnal toxicity, we performed *in vitro* experiments using murine hepatoma Hepa1-6 cells. CuCl_2_ treatment resulted in a dose-dependent inhibition of Hepa1-6 cells viability (Fig. 4A). To investigate the involvement of clock genes, we measured their expression levels after Cu treatment. We observed an upregulation of *Ciart*, *Cry2*, and *Per1* following treatment of Hepa1-6 cells with 500 µM Cu (Fig. 4B). Recent studies reported that clock genes, such as *Per1* and *Per2*, play a role in hepatic injury in mice [29, 30]. Therefore, we hypothesized that the clock genes *Ciart*, *Cry2*, and *Per1* may influence cell viability in response to Cu toxicity in Hepa1-6 cells.

### Contribution of *Ciart*, *Cry2*, and *Per1* against Cu toxicity

**Fig. 5:**
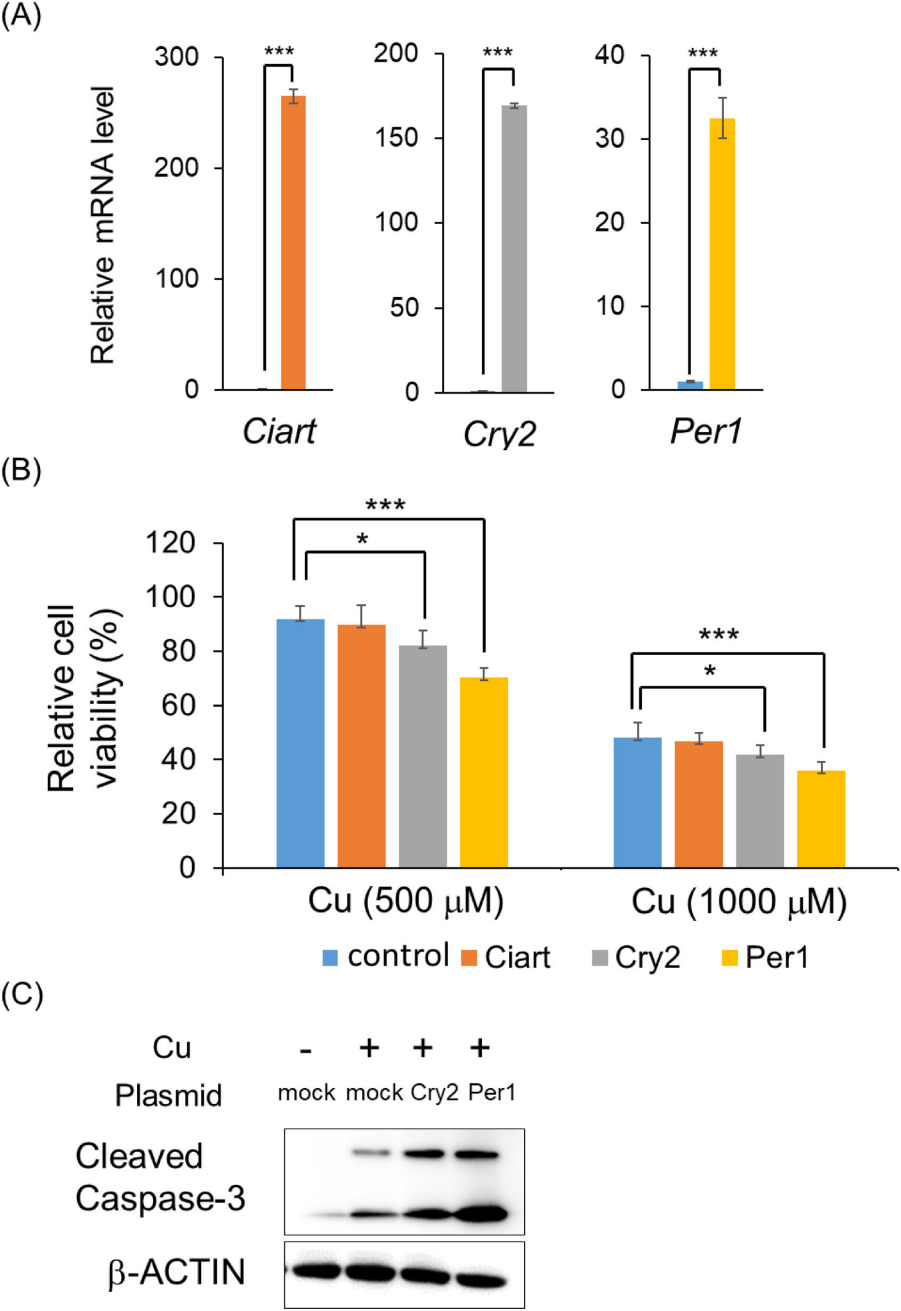
Influence of *Ciart*, *Cry2*, and *Per1* on Cu-induced toxicity in Hepa1-6 cells (A) Hepa1-6 cells were plated at a density of 500 000 cells per 35-mm dish. After 24 h of cell seeding, cells were transfected with 1 µg of plasmids (control, *Ciart*, *Cry2*, or *Per1*). The expression levels of each gene were measured by RT-PCR 24 h after the transfection. ***P < 0.001 versus control (n = 4). (B) Hepa1-6 cells were seeded at a density of 10 000 cells into 96-well plates and transfected with 50 ng plasmids (control, *Ciart*, *Cry2*, or *Per1*) after 3 h of seeding. After 24 h of transfection, each cell was treated with 500 or 1 000 µM of Cu, followed by the measurement of Hepa1-6 cell viability 24 h after the treatment. Data are plotted as mean ± S.D. of groups *P < 0.05 and ***P < 0.001 (n = 6). (C) Hepa1-6 cells were plated at a density of 500 000 cells per 35 mm dish and transfected 1 µg plasmids (control, *Cry2*, or *Per1*) after 24 h of seeding. After 24 h of cell transfection, they were treated with 500 µM Cu for 24 h. We extracted each protein using RIPA buffer and detected cleaved caspase-3 and β-ACTIN by Western blot analysis.

To evaluate the effects of *Ciart*, *Cry2*, and *Per1* against Cu-induced toxicity in Hepa1-6 cells, we conducted transfection experiments. Transfection with *Ciart*, *Cry2*, and *Per1* upregulated the expression of each gene (Fig. 5A). Overexpression of *Cry2* and *Per1* accelerated Cu-induced toxicity in Hepa1-6 cells, whereas overexpression of *Ciart* did not modulate Cu-induced cell viability (Fig. 5B). As Cu is known to induce apoptosis, we additionally evaluated this effect [15, 31]. Treatment with Cu induced the expression of cleaved caspase-3, an indicator of apoptosis, in Hepa1-6 cells (Fig. 5C). Overexpression of *Cry2* and *Per1* accelerated apoptosis. These results indicate that *Cry2* and *Per1* are associated with apoptosis-induced cell death.

### Influence of Cu homeostasis

**Fig. 6:**
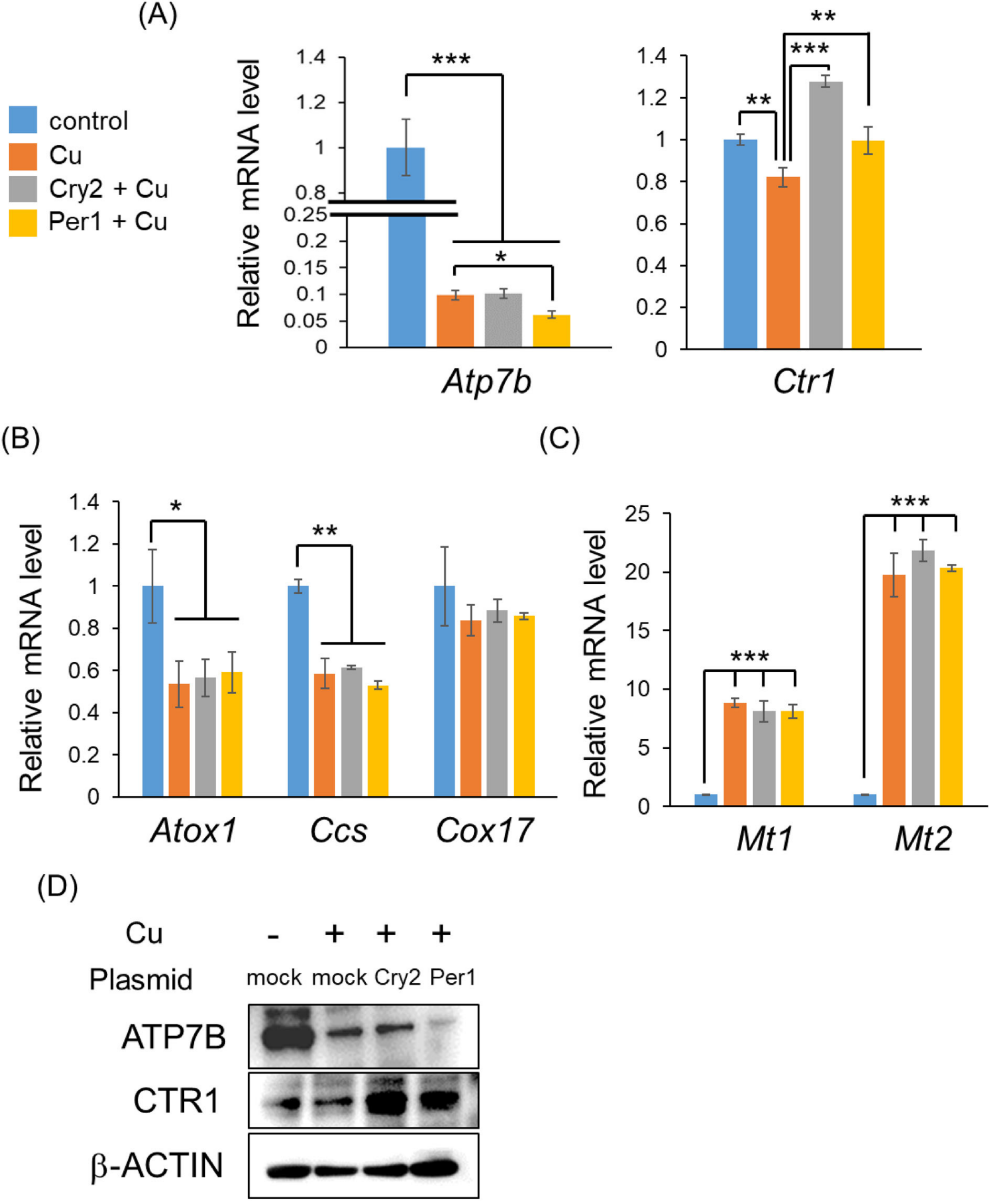
Involvement with Cu homeostasis under *Cry2*, or *Per1* overexpression in Hepa1-6 cells Hepa1-6 cells were plated at a density of 500 000 cells per 35 mm dish and transfected with 1 µg plasmids (control, *Cry2*, or *Per1*) after 24 h of seeding. After 24 h of cell transfection, cells were treated with 500 µM Cu for 24 h. (A–C) The expression levels of genes related to Cu homeostasis (A: transporter, B: chaperone, C: metallothionein) were measured by RT-PCR 24 h after transfection. *P < 0.05, **P < 0.001, and ***P < 0.001 (n = 4). (D) ATP7B, CTR1, and β-ACTIN were detected by Western blot analysis, as described in Fig. 5C.

We measured the expression of genes related to Cu homeostasis, including transporters, chaperones, and metallothionein (MT), to determine their association with *Cry2* and *Per1*. Treatment with Cu significantly downregulated the expression levels of *Atp7b* (Cu exporter) and *Ctr1* (Cu importer) in Hepa1-6 cells (Fig. 6A). Moreover, the overexpression of *Cry2* and *Per1* significantly increased the expression level of *Ctr1* whereas the overexpression of *Per1* significantly decreased the expression of *Atp7b* (Fig. 6A). This result was confirmed at the protein level using immunoblotting (Fig. 6D). Although Cu treatment regulated Cu chaperones (*Antioxidant 1 Copper Chaperone [Atox1]* and *copper chaperone for superoxide dismutase [Ccs]*) and *Mt1/2*, no significant change was observed upon overexpression of *Cry2* and *Per1* (Fig. 6B and 6C). These results suggest that *Cry2* and *Per1* may accelerate Cu accumulation in the liver.

### Influence of clock genes (*Ciart*, *Cry2*, and *Per1*) and Cu homoeostasis (*Atp7b*, *Ctr1*, and accumulation) against Cu toxicity in mice

**Fig. 7:**
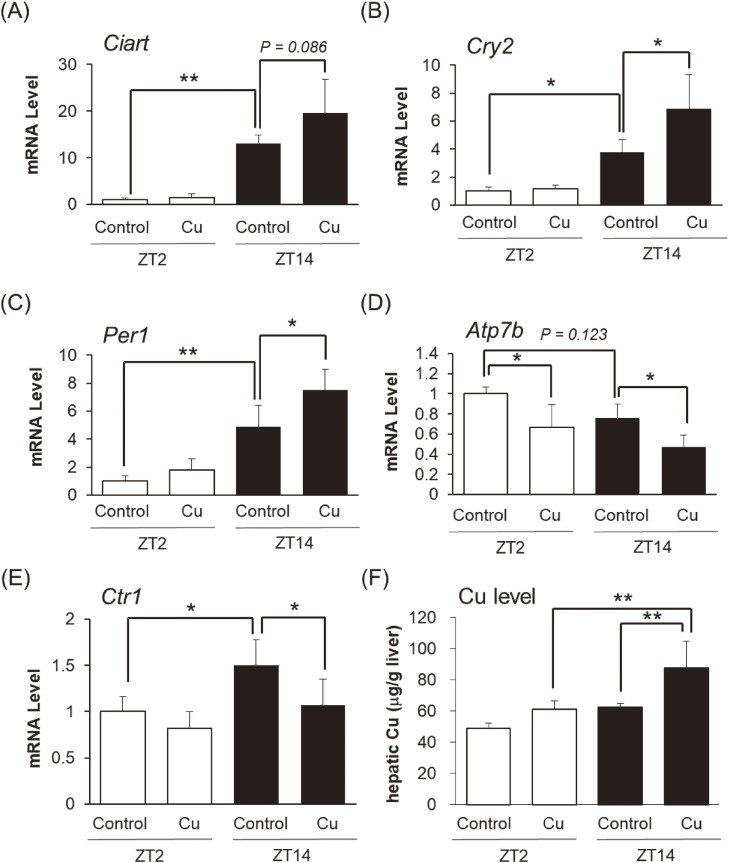
Effect of clock genes and transporters against Cu-repeated administration for 5-weeks in the liver The animals, treated as described in Fig. 2, were euthanized 72 h after intraperitoneal injection, and the livers were harvested. (A–E) Panels indicate *Ciart*, *Cry2*, *Per1*, *Atp7b*, *and* *Ctr1* in the liver, respectively. (F) Hepatic Cu levels were determined by atomic absorption spectrometry. Data are plotted as mean ± S.D. of groups *P < 0.05, **P < 0.01, and ***P < 0.001.

We evaluated expression levels of clock gene (*Ciart*, *Cry2*, and *Per1*) and Cu transporter (*Atp7b* and *Ctr1*) by administration with Cu in mice (Fig. 7). We found that expression levels of *Ciart*, *Cry2*, and *Per1* (Fig. 7A, 7B, and 7C) were higher in the dark phase (ZT14) than that of light phase (ZT2). Cu administration at ZT2 tended to increase each level, whereas Cu administration at ZT14 significantly upregulated *Cry2*, and *Per1* in the liver compared to the control group at ZT14 (Fig. 7B and 7C). Along with the clock genes, we measured Cu transporter (*Atp7b* and *Ctr1*) (Fig. 7D and 7E). We found that *Atp7b* expression level tend to decrease in the dark phase than that of light phase (Fig. 7D, *P* = *0.123*). Cu administration at ZT2 and ZT14 significantly downregulated *Atp7b* in the liver (Fig. 7D). In addition, we demonstrated that *Ctr1* expression level increased in the dark phase than that of light phase (Fig. 7E). Cu administration at ZT2 tended to decrease *Ctr1* level, whereas Cu administration at ZT14 significantly downregulated *Ctr1* in the liver compared to the control group at ZT14 (Fig. 7E). Finally, we measured Cu accumulations in the liver (Fig. 7F). We demonstrated that most of the Cu was excreted after 72 h at ZT2 and ZT14, respectively. On the other hand, Cu accumulation was still left in the group administrated with Cu at ZT14. These results suggest that Cu administration at ZT14 potentiated Cu-induced toxicity through increasing Cu accumulation time in the liver.

## Discussion

Cu homeostasis is known to be controlled by multiple genes [32]. The majority of Cu is imported into mammalian cells through *Ctr1*, and Cu is passed to intracellular chaperone proteins, such as *Ccs*, which carry Cu through the cytoplasm. *Cox17* is a key mitochondrial Cu chaperone protein responsible for the delivery of Cu ions to the mitochondria. Another Cu chaperone protein, *Atox1*, delivers Cu to the Cu-transporting ATPases *Atp7a* and *Atp7b*, which pump Cu into both the secretory and export pathways. MT is a major Cu-binding protein that protects against Cu-induced toxicity [33]. Disruption of Cu homeostasis, whether by Cu overdose or suppression of Cu-related genes, can lead to Cu-induced toxicity, characterized by apoptosis, cell cycle arrest, inflammation, and oxidative stress, in several tissues such as the liver, kidney, brain, and spleen [34–36]. Since the liver is the first site where Cu deposits after entering the bloodstream, and Cu regulation is controlled mainly by the liver, chronic Cu toxicity mainly damages this organ [28].

We have previously demonstrated that Cu-induced sensitivity differed with changing administration times in ICR mice [18]. In this study, we confirmed that the diurnal susceptibility to Cu was conserved between ICR and C57BL/6J mice (Supplementary Fig. S2). This result aligns with our previous findings, which evidenced no difference in Cu chronotoxicity among the three strains (C57BL/6J, ICR, and Balb/c) [16]. Moreover, the diurnal susceptibility to Cu toxicity was observed both with a single and a repeated administration (Fig. 1). Therefore, there was no strain or administration time difference in the diurnal variation in Cu toxicity. Moreover, we demonstrated that diurnal variation of Cu-induced lethal toxicity (8-week administration) and hepatic injury (5-week administration) was confirmed. However, since hepatic injury was mild (Fig. 2 and Fig. 3), it is unclear whether Cu-induced lethal toxicity is occurred through hepatic injury or not. We also evaluated the Cu-induced renal toxicity in mice and found that Cu-induced renal toxicity at ZT14 was stronger than that of ZT2 measured by malondialdehyde, *Il-6* expression level, and morphology (Supplementary Fig. S4). These results suggest that Cu-induced lethal toxicity was associated with multi tissues-induced damage. Further investigation is needed to focus on other tissues such as brain and spleen in the future.

One possible explanation for the diurnal variation in Cu toxicity is the involvement of clock genes. Circadian rhythms are endogenous oscillators that regulate 24-h behavioral and physiological processes, and clock genes play a crucial role in this regulation [37]. At the molecular level, clock gene expression is regulated by BMAL1, CLOCK, and NPAS2. These proteins form heterodimers and function as transcriptional activators. Activation of BMAL1/NPAS2 or BMAL1/CLOCK increases the protein levels of repressors, such as CIART, CRY1/2, and PER1/2/3, which in turn inhibit their own transcription by binding to the BMAL1/NPAS2 or BMAL1/CLOCK heterodimer. Diurnal variation in these clock genes was confirmed in the livers of C57BL/6J male mice (Supplementary Fig. S5). Various studies have suggested that clock genes are involved in the production toxic factors. For example, *Cry1a*, *Cry2a*, and *Per2* regulate oxidative status in zebrafish [38]. *Per1* and *Per2* regulate radiosensitivity in mice [39]. *Clock* knockout mice were found to accelerate acetaminophen-induced hepatic injury [40]. Therefore, it is reasonable to focus on clock genes as potential regulators of Cu-induced toxicity. We found that Cu treatment of Hepa1-6 cells upregulated the expression levels of *Ciart*, *Cry2*, and *Per1*, with well-known high levels in the dark phase (Supplementary Fig. S5 and Fig. 7). These results suggest that the three clock genes act as toxic factors induced by Cu treatment. Overexpression of *Cry2* and *Per1* potentiated the Cu-induced inhibition of Hepa1-6 cells viability, whereas overexpression of *Ciart* did not affect cell viability (Fig. 5B). Although it is unclear how *Ciart* is upregulated by treatment with Cu, *Cry2* and *Per1* are key clock genes associated with Cu-induced diurnal variation. This hypothesis was supported by our *in vivo* experiment, since *Cry2* and *Per1* were also induced by the administration of Cu at ZT14 (Fig. 7).

Elevated levels of cleaved caspase-3 are important markers of apoptosis. Oxidative stress and inflammation disturb mitochondrial homeostasis by increasing its membrane permeability. As a result, cytochrome C is released and, by activating cleaved caspase-3, the apoptotic pathway is initiated [41]. Elevated levels of cleaved caspase-3 have been reported following Cu administration [42]. Moreover, the apoptotic events induced by Cu promote inflammation in mice [42]. We found that the expression levels of *Tnfα* and *Il-6* after Cu injection were significantly higher at ZT14 than at ZT2 (Fig. 2C and 2D); additionally, Cu treatment increased the level of cleaved caspase-3, with the overexpression of *Cry2* and *Per1* in Hepa1-6 cells accelerating this effect (Fig. 5C). Therefore, Cu-induced diurnal toxicity may be attributed to a difference in apoptosis.

To investigate the molecular mechanisms by which *Cry2* and *Per1* regulate Cu-induced toxicity, we measured the mRNA expression of seven genes (*Atp7b*, *Ctr1*, *Atox1*, *Ccs*, *Cox17*, *Mt1*, and *Mt2*) (Fig. 6A, 6B, 6C). We found that *Atp7b* was significantly downregulated by the overexpression of *Per1*. In the liver, *Atp7b* transports Cu from the cytosol into the lumen of the Golgi network for incorporation into ceruloplasmin [43]. *Atp7b* is also required to export Cu excess from the liver. A knock out mice model of *Atp7b* was authorized for the Wilson disease model, as Cu accumulates [44]. Cu accumulation induces marked changes in the liver structure and function. Moreover, we found that *Ctr1* was significantly upregulated by the overexpression of *Cry2* and *Per1*. *Ctr1* is an ATP-dependent transported to the cell membrane. *Ctr1* expression in the liver is regulated at both transcriptional and translational levels, with a lack of Cu inducing *Ctr1* gene expression, whereas Cu overdose downregulates *Ctr1* expression [45–47]. As *Atp7b* and *Ctr1* play important roles in Cu transport and are regulated by the overexpression of *Cry2* and *Per1*, we concluded that *Cry2* and *Per1* regulate Cu-induced apoptosis through the modulation of Cu transporters. In addition, our present data demonstrated that overexpression of *Per1* with Cu downregulated *Atp7b* levels compared to Cu, while overexpression of *Cry2* was comparable to Cu. Moreover, although overexpression of *Cry2* and *Per1* with Cu upregulated *Ctr1* level compared to Cu, upregulation level between overexpression of *Cry2* and *Per1* was different. These data suggest that the potentiation of Cu toxicity by *Cry2* and *Per1* is different. To support this hypothesis, we found that overexpression of *Per1*, not *Cry2*, downregulated *Atp7b* expression level (around 35%) in Hepa1-6 cells (Data not shown). Since *Atp7b* knock out mice was reported to accumulate Cu and induce hepatic injury [48], related phenomenon may occur by overexpression of *Per1*. Moreover, we found that overexpression of *Cry2*, not *Per1*, upregulated *Ctr1* expression level (around 20%). Since *Ctr1* was associated with Cu incorporation, overexpression of *Cry2* may contribute to enhance Cu uptake. To elucidate the association with Cu transporters and *Cry2*/*Per1* in detail, future work should be conducted to use knock out mice and/or knock out cell line of *Cry2* and *Per1*.

## Conclusion

The present study demonstrated that Cu-induced chronotoxicity is conserved across multiple factors, including mouse strain, administration times, and toxicity levels. Moreover, Cu treatment disrupted specific clock genes such as *Ciart*, *Cry2*, and *Per1* both *in vivo* and *in vitro*. Additionally, we established that overexpression of *Cry2* and *Per1* exacerbates Cu-induced hepatotoxicity through the regulation of the Cu transporters (*Atp7b* and *Ctr1*) in Hepa1-6 cells. These results suggest that clock genes induction by Cu exposure modulate Cu toxicity. The Cu concentration we used are high concentration compared to workplace in human except major accident. However, our present findings are particularly relevant for shift workers in the steel industry, who may handle Cu in both light and dark phases of the day. The impacts of our results are significant for the safety and self-protection of these workers, given that Cu exposure can alter clock gene levels, during the light and dark phases of the day. While further investigation is needed to fully elucidate the mechanisms by which *Cry2* and *Per1* modulate Cu transport, our present study highlights the importance of considering injection timing in basic research and underscore the need for careful self-protection measures for people working in the steel industry.

## Abbreviations

ALT: alanine aminotransferase
ANOVA: analysis of variance
AST: aspartate aminotransferase
Atox1: antioxidant 1 Copper Chaperone
ATP7B: ATPase Copper Transporting Beta
Ccs: copper chaperone for superoxide dismutase
Ciart: circadian associated repressor of transcription
Ctr1: copper uptake protein 1
Cu: copper
Cry: cryptochrome
DMEM: dulbecco’s modified Eagle’s medium
H&E: hematoxylin and eosin
Il-6: Interleukin-6
Tnfα: tumor necrosis factor alpha
MT: metallothionein
Npas2: neuronal PAS domain protein 2
Per2: period2
ZT: zeitgeber time

## Supplementary information

**Additional file 1:** **Supplemental Figure S1.** Process of plasmid construction. **Supplemental Figure S2.** Diurnal variation in Cu-induced mortality. **Supplemental Figure S3.** Effect of body weight against Cu repeated injection for 5 weeks. **Supplemental Figure S4.** Cu injection at ZT14 increased renal malondialdehyde and worsened the renal morphology by H&E staining. **Supplemental Figure S5.** Spatiotemporal expression of nine clock genes in the liver.

**Additional file 2:** **Supplemental Table S1.** Primer lists for qPCR.

## Declarations

### Availability of data and material

The datasets used and/or analyzed during the current study are available from the corresponding author on reasonable request.
