# Supplementary material for: Copper-induced diurnal hepatic toxicity is associated with Cry2 and Per1 in mice
Source: Environ Health Prev Med. 2023 Dec 13;28:78. doi: 10.1265/ehpm.23-00205 (PMC10739358; doi:10.1265/ehpm.23-00205)

**Supplementary Information**

**Materials and Methods**

*Experimental protocol in lethal toxicity test*

Seven-week-old male C57BL/6J mice were assigned to six groups of five animals and were administrated with 9.0 mg (66.8 μmol Cu) /kg CuCl_2_ (Fujifim Wako Pure Chemical) intraperitoneally (i. p.) at six different time points per group (clock time; 10:00, 14:00, 18:00, 22:00, 2:00, or 06:00), corresponding to zeitgeber time (ZT); ZT2, ZT6, ZT10, ZT14, ZT18, or ZT22. During the dark period (ZT14, ZT18, ZT22), mice were injected with Cu under red light. Mortality was monitored for fourteen days after injection.

*Experimental protocol of the spatiotemporal expression test*

Seven-week-old male C57BL/6J mice (n=3–5) were euthanized using carbon dioxide, and liver samples were collected at ZT2, ZT6, ZT10, ZT14, ZT18, or ZT22. The obtained samples were stored at -80°C.

*Plasmid construction*

The progress of plasmid construction is described in Supplementary Figure S1. The cDNA of the coding region of the *Ciart* (NM_001033302) gene was amplified from the liver of male C57BL/6J mice using the Expand High Fidelity PCR System (Roche, Basel, Switzerland), and then subcloned into a pANT vector (NIPPON Gene, Tokyo, Japan) and named pANT-Ciart. The *Bmal1* fragment was amplified with specific primer sets (forward primer [5′- ATGGATTCTCCATCTAGTGTTTCTTC-3′] and reverse primer [5′- AATCTAGATTAGGGCTGAGGATCCGGAG-3′] using KOD-one (Toyobo, Osaka, Japan). The amplified *Ciart* fragment was restricted with *Xba*I at 37°C for 1 h. pcDNA3.1-neo was digested with *Eco*RV and *Xba*I at 37°C for 1 h. The *Ciart* fragment was subcloned into the pcDNA3.1-neo fragment using Ligation high Ver.2 (Toyobo); this construct was named pcDNA3.1-neo-Ciart.

The cDNA of the coding region of the *Cry2* (NM_0099963) gene was amplified from the liver of male C57BL/6J mice using the Expand High Fidelity PCR System, and then subcloned into a pANT vector (NIPPON Gene, Tokyo, Japan) and named pANT-Cry2. The *Cry2* fragment was amplified with specific primer sets (forward primer [5′- ATGGCGGCGGCTGCTGTG-3′] and reverse primer [5′-AATCTAGATCAGGAGTCCTTGCTTGCTGG-3′] using KOD-one. The amplified *Cry2* fragment was restricted with *Xba*I at 37°C for 1 h. pcDNA3.1-neo was digested with *Eco*RV and *Xba*I at 37°C for 1 h. The *Cry2* fragment was subcloned into the pcDNA3.1-neo fragment using Ligation high Ver.2; this construct was named pcDNA3.1-neo-Cry2.

The cDNA of the coding region of the *Per1* (NM_011065) gene was amplified from the liver of male C57BL/6J mice using the Expand High Fidelity PCR System, and then subcloned into a pANT vector and named pANT-Per1-F1 (192-1581), pANT-Per1-F2 (1563-2842) and pANT-Per1-F3 (2824-4107). Two fragments (F1 and F2) of *Per1* were amplified with specific primer sets (F1:forward primer [5′-GACTCACTATAGGGCATGAGTGGTCCCCTAGAAGG-3′] and reverse primer [5′-GTGCGCACTTTATGGCGAC-3′]; F2: forward primer [5′-GTCGCCATAAAGTGCGCAC-3′] and reverse primer [5′- GTAGGGCTGGACCATTGCT -3′]) using KOD-one. The pANT-Per1-F3 inverse polymerase chain reaction (PCR) fragment was amplified using a specific forward primer (5′- AGCAATGGTCCAGCCCTAC -3′) and reverse primer (5′- GCCCTATAGTGAGTCGTATTAC-3′). The *Per1* fragments (F1 and F2) were subcloned into the pANT-Per1-F3 fragment using the In-Fusion HD Cloning Kit (Takara Bio, Shiga, Japan); this construct was named pANT-Per1.The *Per1* fragment was amplified with specific primer sets (forward primer [5′- ATGCTAGCATGAGTGGTCCCCTAGAAGGG-3′] and reverse primer [5′- GGAAGCTTCCAAAATGGAGTCTAGCTGGTG-3′] using KOD-one. The amplified *Per1* fragment and pcDNA3.1-neo were digested with *NheI* and *HindIII* at 37°C for 1 h. The *Per1* fragment was subcloned into the pcDNA3.1-neo fragment using Ligation high Ver.2; this construct was named pcDNA3.1-neo-Per1.

*Measurement of malondialdehyde levels in the kidney*

Total malondialdehyde (MDA) levels and total antioxidant capacity in the kidney were examined using a colorimetric thiobarbituric acid reactive substances microplate assay kit (Oxford Biochemical Research, Oxford, MI) according to the manufacturer's protocols.

**Supplementary Figure Legends**

Supplemental Figure S1. Process of plasmid construction

Panels (A), (B), (C), (D), (E), (F), (G), and (I) indicate the process of pANT-Ciart, pcDNA-Neo-Ciart, pANT-Cry2, pcDNA-Neo-Cry2, pANT-Per1-F1, pANT-Per1-F2, pANT-Per1-F3, pANT-Per1and pcDNA-Neo-Per1 plasmid construction, respectively.

Supplemental Figure S2. Diurnal variation in Cu-induced mortality

Male C57BL/6J mice (n=5) were intraperitoneally injected with 9.0 mg/kg CuCl_2_ at ZT2, ZT6, ZT10, ZT14, ZT18, or ZT22. Survival was recorded seven 14-days after the injection in each group (A). Mean survival time (MST, expressed as days) was estimated until 14-days after the injection using Kaplan–Meier analysis (B).

Supplemental Figure S3. Effect of body weight against Cu repeated injection for 5 weeks

Male C57BL/6J mice were injected i.p. with 6.08 mg/kg CuCl_2_ twice per week at ZT2 or ZT14 through the 5 weeks.

Supplemental Figure S4. Cu injection at ZT14 increased renal malondialdehyde and worsened the renal morphology by H&E staining.

Male C57BL/6J mice were injected i.p. with 6.08 mg/kg CuCl_2_ twice per week at ZT2 or ZT14 through the 5 weeks. Mice were euthanized 72 hours after the final Cu injection. Panel (A) indicated renal malondialdehyde level. Panel (B) indicated renal *Il-6* levels. **P*<0.05 and ***P*<0.001. Kidney specimens were fixed, processed using standard methods, and stained with H&E (C). Black arrows show injury in the renal tubules.

Supplemental Figure S5. Spatiotemporal expression of nine clock genes in the liver

Relative expression of nine clock genes (*Bmal1, Ciart, Clock. Cry1, Cry2, Npas2, Per1, Per2*, and *Per3)* in the liver from ZT2 to ZT22.


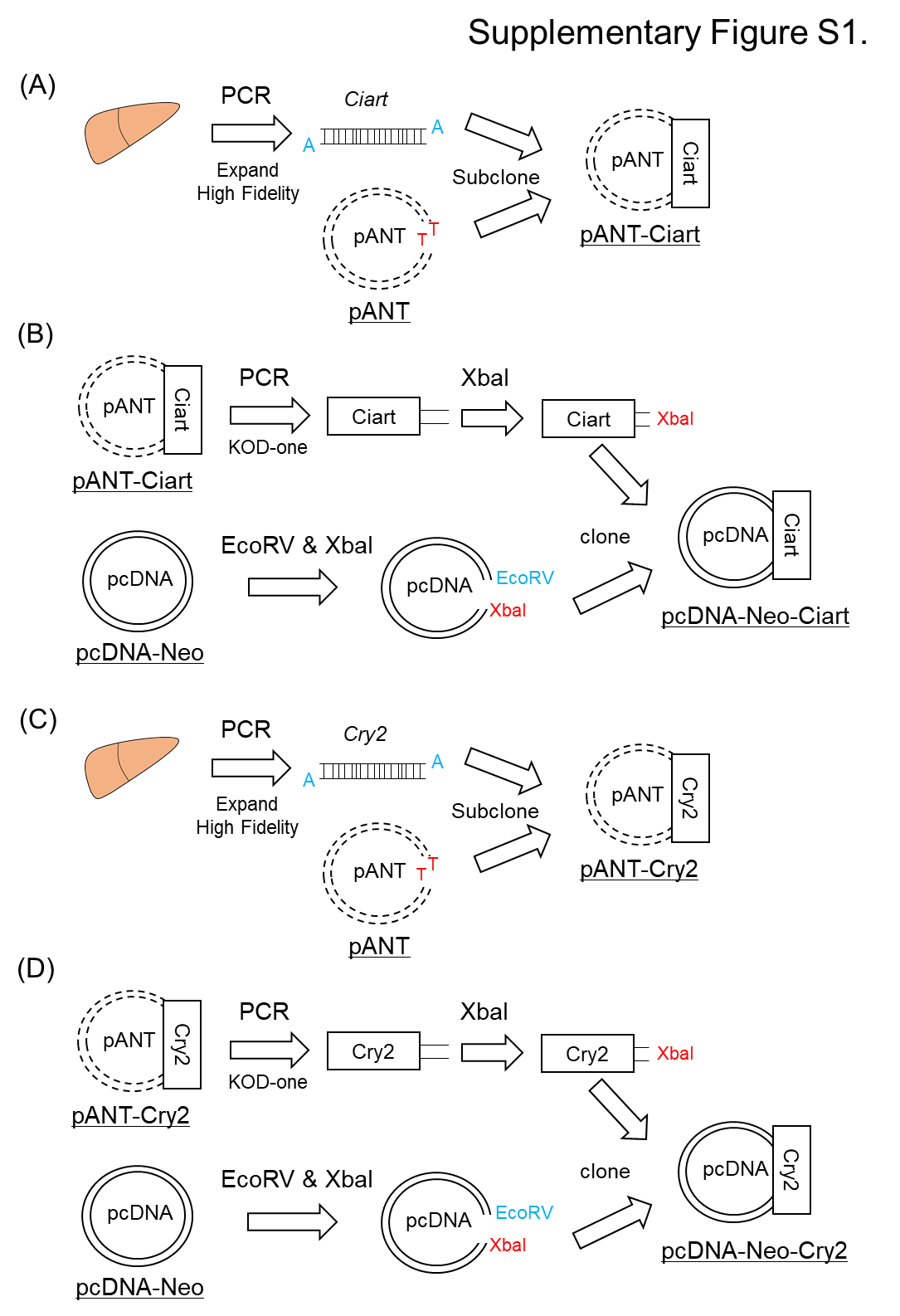


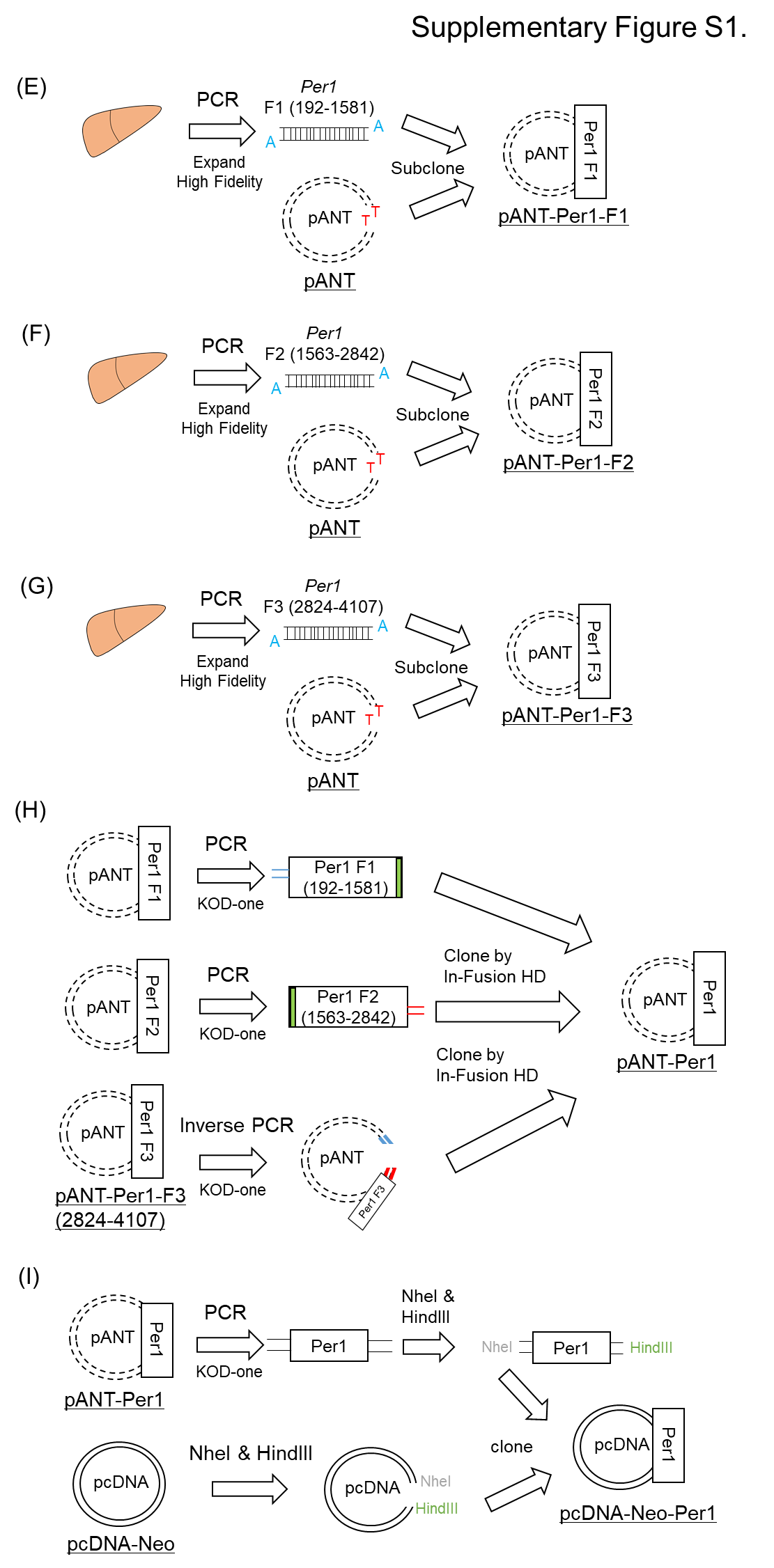


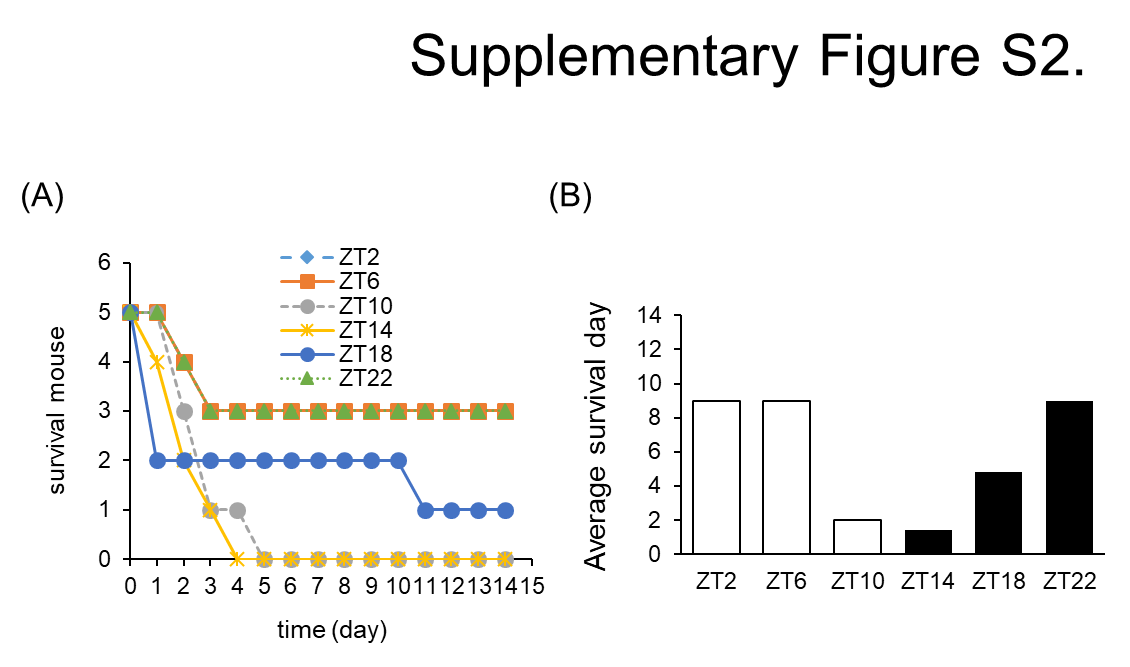


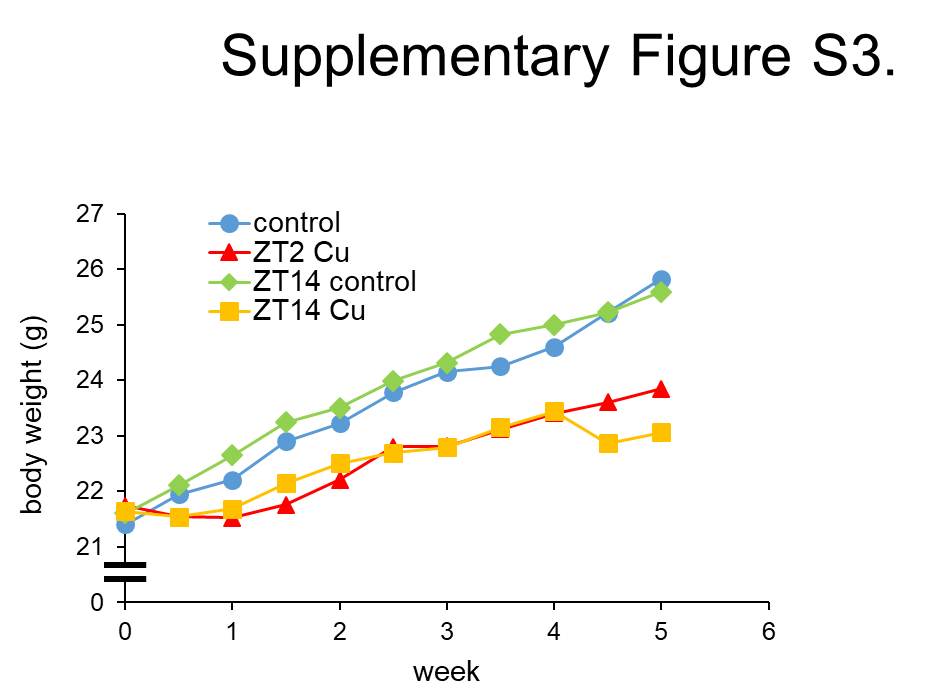


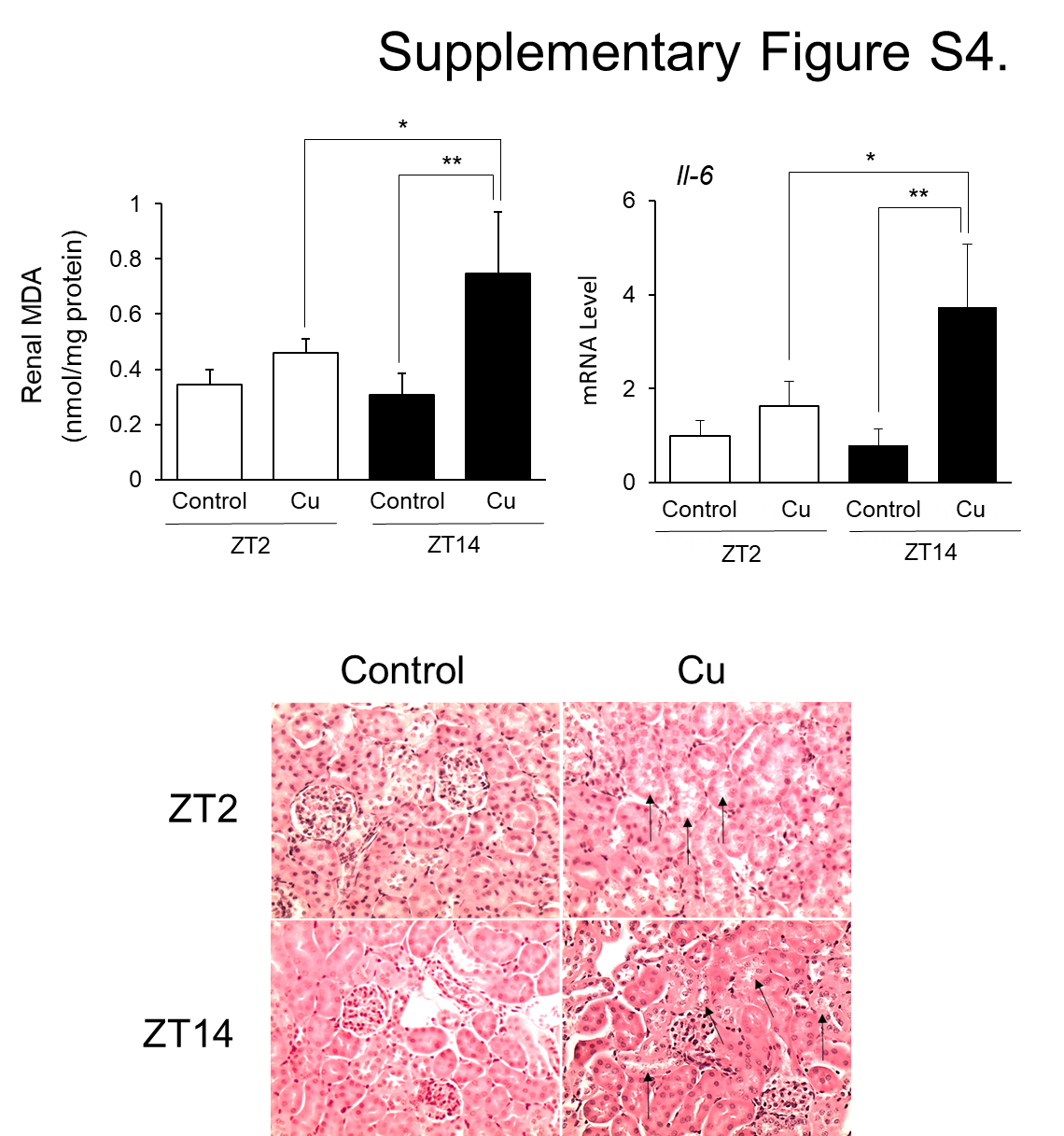


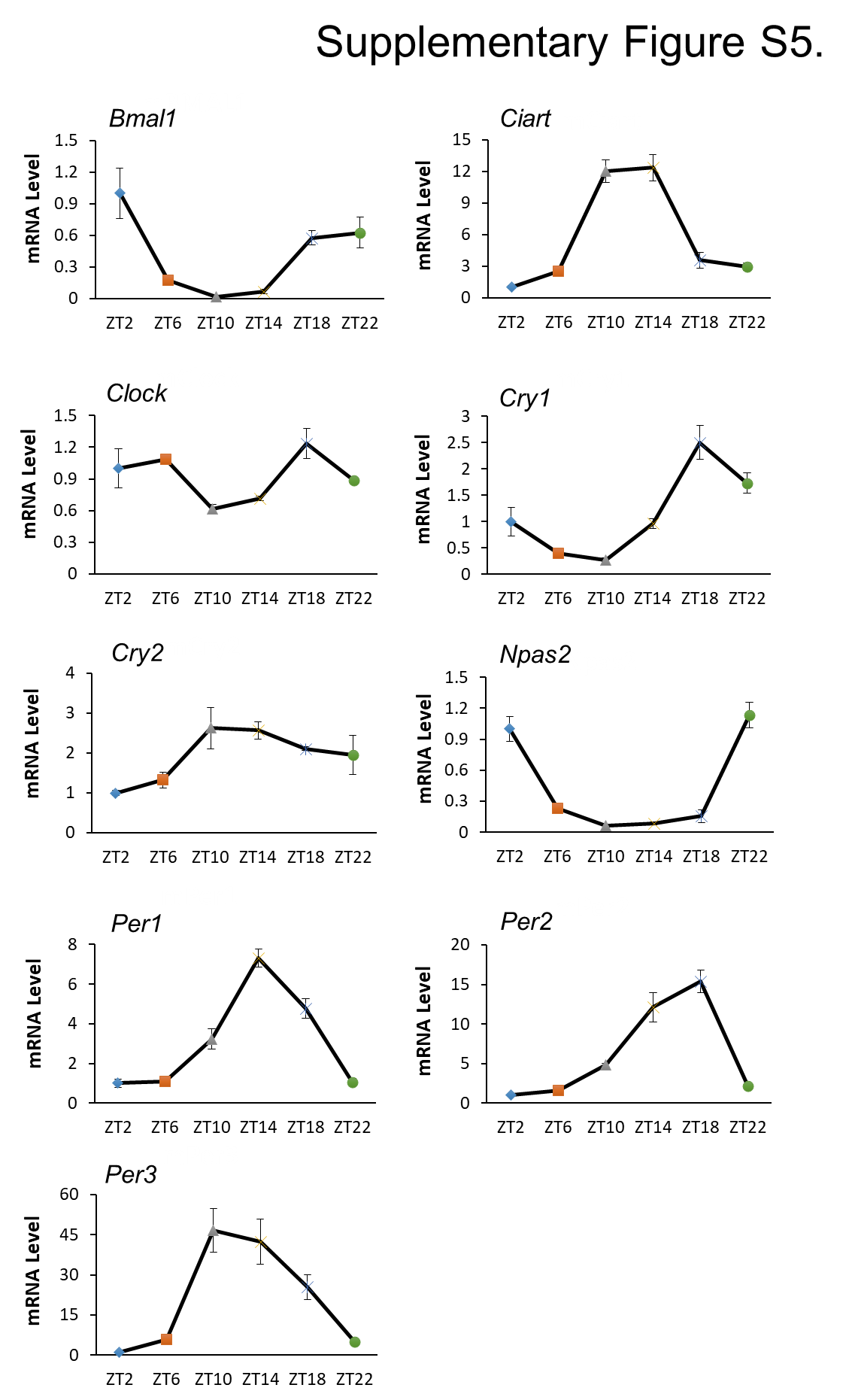

Supplement: Supplementary file 1 — Additional file 1: Supplemental Figure S1. Process of plasmid construction. Supplemental Figure S2. Diurnal variation in Cu-induced mortality. Supplemental Figure S3. Effect of body weight against Cu repeated injection for 5 weeks. Supplemental Figure S4. Cu injection at ZT14 increased renal malondialdehyde and worsened the renal morphology by H&E staining. Supplemental Figure S5. Spatiotemporal expression of nine clock genes in the liver. [file ehpm-28-078-s001.doc]
