# Supplementary material for: Copper-induced diurnal hepatic toxicity is associated with Cry2 and Per1 in mice
Source: Environ Health Prev Med. 2023 Dec 13;28:78. doi: 10.1265/ehpm.23-00205 (PMC10739358; doi:10.1265/ehpm.23-00205)
Supplement: Supplementary file 2 — Additional file 2: Supplemental Table S1. Primer lists for qPCR. [file ehpm-28-078-s002.docx]

Supplemental Table S1. Primer lists for qPCR

| *Gene* | Forward primer | Reverse primer |
| --- | --- | --- |
| *Il-6* (NM_031168) | GAAATGGATGCTACCAAAC | TACTCCAGGTAGCTATGGTACT |
| *Tnfα* (NM_013693) | ACACTCAGATCATCTTCTCAAAATTCG | GTGTGGGTGAGGAGCACGTAGT |
| *Bmal1* (NM_007489) | TTCTCCAGGAGGCAAGAAGA | TTGCTGCCTCATCGTTACTG |
| *Ciart* (NM_001033302) | ATAGCTGCCCAGAAGTCATCC | TCTAGGAGAGGTGCAGGAGAAG |
| *Clock* (NM_007715) | TGCCAGCTCATGAAAAGATG | CGCTGCTCTAGCTGGTCTTT |
| *Cry1* (NM_007771) | AGTTCCCCTCCCCTTTCTCTT | GGGTTCCCTTCCATTTTGTCA |
| *Cry2* (NM_0099963) | GCAAAGGACTACGGCTCCAC | ATCTTCCAGAGATTGCAGTAGGAAC |
| *Npas2* (NM_008719) | GAGGCAGCTTGAACCCAAAGG | GTCAAAGCCATTACAGGAGGGG |
| *Per1* (NM_011065) | GGGAGCTCAAACTTCGACTG | TCGGATGTGATAGCTCCAA |
| *Per2* (NM_011066) | TAGAATCCCTCCTGAGAAGAGG | AGAATAATCGAAAGGCTGTCCAC |
| *Per3* (NM_011067) | TGGCTGCAGGAGGATTTAAG | TGCTGTGCTTAGCAGTGGAC |
| *Mt1* (NM_013602) | TTCACCAGATCTCGGAATGG | GAGGTGCACTTGCAGTTCTTG |
| *Mt2* (NM_008630) | CCTGCAAATGCAAACAATGC | CACTTGTCGGAAGCCTCTTTG |
| *Atp7b* (NM_007511) | CGAACTCCGAACGGCTGTAG | TGGCACAGAATTCCCAGATTTGAAG |
| *Ctr1* (NM_175090) | CCGGTTTGGTAATCAATACACCTG | GACCCTCTCGGGCTATCTTG |
| *Atox1* (NM_009720) | CCGTCTCCAGAGTCCTCAAC | TGTCTGAGCTGTGCTCAGAG |
| *Ccs* (NM_016892) | GGGGACCATTTTAACCCTGATGG | TCTATCCGGAAGGTAGCTCGG |
| *Cox17* (NM_001017429) | AGGAGAAGAAGCCACTGAAGC | GGCTTCAATGAGATGTCCACAG |
| *β-actin* (NM_007393) | GCAACGAGCGGTTCCG | CCCAAGAAGGAAGGCTGGA |
